# Improving Physical Fitness and Cognitive Functions in Middle School Students: Study Protocol for the Chinese Childhood Health, Activity and Motor Performance Study (Chinese CHAMPS)

**DOI:** 10.3390/ijerph15050976

**Published:** 2018-05-14

**Authors:** Zhixiong Zhou, Shanshan Dong, Jun Yin, Quan Fu, Hong Ren, Zenong Yin

**Affiliations:** 1 Institute for Sport Performance and Health Promotion, Capital University of Physical Education and Sports, Beijing 100191, China; sec126@126.com (S.D.); yinjun@cupes.edu.cn (J.Y.); fuquan@cupes.edu.cn (Q.F.); 2 School of Sport Sciences, Beijing Sport University, Beijing 100084, China; renhong5939@sina.com; 3 Department of Kinesiology, Health and Nutrition, The University of Texas at San Antonio, San Antonio, TX 78249, USA

**Keywords:** cardiorespiratory fitness, moderate to vigorous physical activity, physical function training, after school program

## Abstract

*Background*: Sedentary lifestyles and their associated harmful consequences are public health concerns that impact more than half of the world’s youth population in both developed and developing countries. *Methods*: The Chinese Childhood Health; Activity and Motor Performance Study (Chinese CHAMPS) was a cluster randomized controlled trial to modify school physical activity policies and the physical education (PE) curriculum; using teacher training and parent engagement to increase opportunities and support students’ physical activity and healthy eating. Using a 2 × 2 factorial design, the study tested the incremental effects of increasing the amount and intensity of physical activity, alongside adding support for healthy eating, on health-related and cognitive function outcomes in Chinese middle school students. *Results*: The intervention was implemented by PE teachers in 12 middle schools in three Chinese cities, with a targeted enrollment of 650 students from August 2015–June 2016. The assessment of the outcomes involved a test battery of physical fitness and cognitive functioning at both baseline and at the end of the intervention. Process information on implementation was also collected. *Discussion*: The Chinese CHAMPS is a multi-level intervention that is designed to test the influences of policy and environmental modifications on the physical activity and eating behaviors of middle school students. It also addresses some key weaknesses in school-based physical activity interventions.

## 1. Introduction

A secular trend of an increasingly sedentary lifestyle has been observed in both developed and developing countries over the past half century [1]. In school-age children, a lack of moderate to vigorous physical activity (MVPA) and excessive time spent engaging in sedentary behavior are the prominent characteristics of a sedentary lifestyle, which affects nearly two thirds of the children in the world according to the World Health Organization [2]. National and international health organizations have recommended that school-age children should participate in at least one hour a day (h/d) of MVPA and reduce time spent engaging in sedentary behavior to benefit physical, cognitive, and emotional growth [2,3]. Furthermore, findings from both cross-sectional [4,5] and longitudinal epidemiological studies [1,6] demonstrate that MVPA and vigorous physical activity (VPA) in particular were favorably associated with aerobic fitness, body composition, and cardiometabolic measures. On the other hand, increased risk for obesity and poor physical fitness in children is linked with excessive time spent in sedentary activities, such as watching TV and homework [1,7,8,9]. An emerging body of literature also suggests that regular participation in physical activity (PA) is associated with better academic achievement, while sedentary children suffer from poor development of cognitive functions [10,11,12]. Physical fitness is believed to be the mediator between PA and the development of cognitive functions in children.

School is where children spend most of their waking hours, and therefore many opportunities exist to promote physical activity and nutrition behaviors by modifying school policies and the school environment [13,14]. Past studies have reported promising results from modifications to the physical education (PE) curriculum and recess, PE teacher training, afterschool PA intervention, and increased engagement by policy makers and parents. However, the recent Cochrane reviews revealed that school-based PA intervention programs had only small effects on the rates of participation in PA and improvements in physical fitness [15] and cognitive functions [11]. Therefore, the need to identify effective and innovative strategies to promote PA in school settings remains relevant.

Increases in physical inactivity and sedentary behavior [16,17,18] have been observed in Chinese school-age children in recent decades, and are associated with declines in physical fitness [19,20] and an upward trend in obesity [16,21]. According to the 2010 National Physical Fitness and Health Surveillance, only 22.7% of Chinese elementary and secondary school students aged 9–18 report being physically active for at least 60 min a day [16]. Another national survey found that nearly one third of elementary and secondary schools failed to implement the national PE recommendations in 2010 [22]. This stipulates that students in grades 1–2, grades 3–9, and grades 11–12 must be offered 4 h, 3 h, and 2 h of PE each week, respectively. The same report also revealed that only 18% of the students met the recommendation of 60 min of MVPA each day, while 40% engaged in less than half an hour of MVPA daily. The Chinese Ministry of Education has made an urgent call for local education officials and school administrators to increases opportunities for PA in Chinese schools [23].

We conducted a randomized control trial (RCT) to test the effects of variations in duration and intensity of PA on physical fitness and cognitive functioning in middle school students, and to document the fidelity of the program implementation for Chinese middle school students. Our study was designed to address some of the key weaknesses in school-based PA intervention research. These include the use of non-RCT designs [15,24], lack of support for policy and environmental changes [24], inadequate PA dose [15], poor teacher training [25], lack of process evaluation [26], poor parent involvement [27], and poor validity of the measurement of PA, sedentary behavior, and cognitive functioning [12,15,25]. Similar challenges have also been reported in PA studies in Chinese schools [21,22,26,28]. With the support of the study schools, we were able to conduct a RCT and to address some of key methodological weaknesses in the current research. To our knowledge, no study of similar design has been reported in the literature. We report the rationale of the intervention programs and study design in this manuscript.

## 2. Methods

Trial registration: ChiCTR-IOR-14005388, The Childhood Health, Activity and Motor Performance Study (Chinese CHAMPS). Approval ID number is 201509003 (15 September 2015).

### Study Design and Sample

The Chinese Childhood Health, Activity and Motor Performance Study (Chinese CHAMPS) is a cluster RCT that included 12 middle schools from three Chinese cities. The three cities were chosen from a list of four cities to represent schools from large (Beijing), medium (Wuhu, Anhui Province), and small (Weifang, Shandong Province) metropolitan regions in China. In each city, four schools with similar student enrollment numbers, student-teacher ratios, and outdoor facilities were recruited with support and facilitation from the City Bureau of Education. We planned to recruit a total of 650 students (an average of 60 students from each school) enrolled in seventh grade. The Chinese middle schools were co-ed and included grades seven (age 12), eight (age 13), and nine (age 14). Assuming a 90% retention rate, a final sample of 540 students would provide sufficient power to detect a small additive effect (i.e., the main effects of the three intervention levels) for the primary study outcomes for a Generalized Estimating Equations (GEEs) model (α ≤ 0.05, 2-sided test) [15,29].

Using a 2 × 2 factorial design, the intervention had four conditions: Arm 1—school physical education intervention (SPE), Arm 2—afterschool program intervention (ASP), Arm 3—SPE + ASP, and Arm 4—control. The primary hypotheses were that (i) students in Arm 1, Arm 2, and Arm 3 would have higher levels of cardiorespiratory fitness (CRF; the primary outcome) than the control students in Arm 4; (ii) students in Arms 1 and 3 would have higher levels of CRF than students in Arm 2; and (iii) students in Arm 3 would have higher levels of CRF than students in Arm 1, at the end of the study. We also hypothesized that there would be significant improvements in other measures of physical fitness and a reduction in body fat percentage and waist circumference in students in Arms 1, 2, and 3, compared to students in Arm 4, at the end of the study (secondary outcome). Finally, we hypothesized that students in Arms 1, 2, and 3 would have higher cognitive functioning scores than students in Arm 4 at the end of the study (secondary outcome).

To be eligible for the study, a school needed to (i) have 80–100 students of both genders enrolled in seventh grade; (ii) be located at least five kilometers apart from other study schools; (iii) agree to the randomization of treatment; and (iv) agree to implement policy and curriculum modifications. After receiving the approval of each school’s principal, the four schools in each city were randomly assigned to one of the four treatment conditions.

Student recruitment was coordinated by the school principals and PE teachers. Parents were informed of the study in announcement posters at the beginning of the school year. All parents received informed consent letters and were asked to indicate if they consented for their children to participate in the study. Signed consent letters were returned to the PE teachers. No incentive was provided for participation in the study. Student eligibility criteria included current enrollment, parental consent, and no physical disability. Students were excluded if they were members of varsity sport teams. The intervention was implemented from August 2015–June 2016 excluding the winter break. Baseline and post-study assessments were conducted immediately before and after the intervention. The study protocol (ChiCTR-IOR-14005388) was approved by the Ethics Committee at the Capital University of Physical Education and Sport.

## 3. Description of Intervention

### 3.1. Theoretical Framework of the Intervention Design

The design of Chinese CHAMPS was informed by the socio-ecological models (SEM) of health promotion [30] and competence motivation theory (CMT) [31] that have been shown to favorably influence physical fitness, PA, diet, and sedentary behaviors in adolescents in RCTs conducted in high-income countries. By recognizing multiple-level influences on student health development, we crafted the intervention to modify school policy and the school environment (teacher training, PE curriculum modifications, and nutrition education) and increase family support, which in turn would increase the students’ exposure and access to MVPA and healthy eating at school and at home. The CMT was used in the design of developmentally-appropriate and physically challenging activities to enhance perceived competence as well as enjoyment of the activities among the students, and to promote movement skill development and physical fitness while engaging students in challenging and novel PA. This was achieved with modification and adaptation of activities to accommodate each student’s levels of skill and physical fitness, and to be fun and novel for reducing boredom and increasing engagement.

### 3.2. Intervention Design

Physical education is compulsory in Chinese schools. Chinese middle schools must implement the national PE standards, which specify the goals, objectives, and basic content for the PE curriculum and subsequent student evaluation [32]. In urban areas, Chinese PE teachers generally have a degree in PE from a two- or four-year college. Physical education teachers are required to develop yearly and monthly curriculum plans and have weekly lesson plans. Middle school students have two periods of PE each week that usually last 45 min each. The Ministry of Education recommends offering two hours of PA weekly in middle schools [33]. Schools are asked but not required to provide recess and afterschool programs to increase participation in PA.

The primary objective of the Chinese CHAMPS study was to test the incremental effect (activity dose) of varied amounts of MVPA and VPA on the study outcomes using a 2 × 2 factorial design. The majority of PA intervention studies have produced only a modest effect on physical fitness and cardiometabolic outcomes in children due to inadequate intervention dose or poor program quality [15,24]. Systematic reviews, however, revealed that high quality interventions that target CRF and additional attributes of physical fitness with a high dose intervention (activity exposure time: 160–200 min of MVPA/week; activity intensity target: an average heart rate of 140 beats/minutes or higher) have shown efficacy (a small to medium effect) in improving physical fitness and in improving body composition in normal weight as well as overweight and obese children [34,35,36]. In addition, observation studies have shown that 15–45 min of daily VPA is associated with improved CRF and a lower body mass index (BMI) in children [37,38,39]. However, no study has systematically tested the effect of varying VPA in school-age children using an RCT design. Based on these findings, we designed our interventions to examine the effect of a varied amount of MVPA and VPA on physical fitness and body composition by modification of the PE curriculum and addition of PA during afterschool hours [24]. The aims were to provide challenging and stimulating activities, and to dedicate 50% of the activity time to VPA during PA sessions. Table 1 shows the intervention dose of MVPA and VPA delivered by each of the treatment conditions in the Chinese CHAMPS.

We incorporated a unique form of physical conditioning based on the principles of physical function training (PFT) in the intervention [40]. Originating from athletic injury rehabilitation, PFT utilizes specially-designed multi-muscle and multi-joint movements to refine fundamental movement skills and neuromuscular coordination, improve physical conditioning, and prevent injuries [41,42]. Due to their popularity with Chinese Olympic athletes, PFT exercises have been introduced to non-elite sport populations including school-age children in China [41]. Utilizing small, portable equipment, the exercises are often designed as circuit-like stations that target different attributes of physical fitness and reduce boredom. The exercises can be easily adapted to levels of individual fitness, which can lead to an improved sense of mastery and enjoyment. The exercises are fun, novel, and challenging, making use of a variety of equipment and quick changes of activity pace. Table 2 shows the functions targeted by PFT and the types of exercises that were used in the intervention program.

The 2 × 2 factorial design was based on two different types of intervention programs in the Chinese CHAMPS study. Table 3 shows the intervention activities.

#### 3.2.1. School Physical Education Policy and Curriculum Modification

The main objective of the SPE intervention was to increase the amount of time in MVPA and VPA during school hours. In addition, nutrition education was introduced to provide students with knowledge of healthy eating. The intervention was implemented by trained PE teachers with the support of a PE assistant. The SPE intervention had three components:

(1) Modifications of school PE policy and PE equipment. The schools agreed to implement a policy to offer three 45 min PE classes a week and added a 15 min recess at mid-morning for each school day. Each intervention school also received portable equipment such as lower hurdles, quick ladders, mini-bands, jump ropes, agility cones, medicine balls, and training cables that were used in the modified PE curriculum.
(2) Physical education curriculum modification. An expert team of PE specialists and researchers redesigned the seventh grade PE curriculum, following the national PE standards [32] and using a games-based approach involving chasing games, ballgames (basketball, volleyball, and soccer), and track and field exercises that were modified to accommodate the different levels of student’s skills and fitness and to motivate participation. The expert team also created a variety of physical fitness exercise stations using aerobic and anaerobic running, jumping, and throwing exercises, all utilizing portable equipment. The designs of the exercise stations were based on the principles of PFT to increase exercise intensity, specificity, and novelty [41,42]. The PE teachers changed the station layouts to target different attributes of physical fitness, adjust activity intensity and volume, create exercise novelty, and increase enthusiasm in their students. The expert team also developed weekly PE lesson plans based on the modified PE curriculum. The PE teachers used these lessons with modifications in planning their daily lessons. To increase MVPA, the expert team developed a 15 min rhythmic aerobic exercise routine for class recess. All students participated in the daily exercise routine in an open space led by a PE teacher. On inclement weather days, the PE teachers presented fitness and nutrition related information to the students based on the Adolescent Fitness and Health Handbook in classrooms. Finally, bi-weekly text messages were sent to the mobile devices (phones or tablets) of the school students using a popular social media app, WeChat (https://www.wechat.com/en/), that provided tips and motivational messages to promote physical activity and healthy eating.
(3) Physical education teacher training. The intervention school PE teachers completed a two-day training on adolescent growth and development, pedagogical methods, and instructional strategies to enhance their knowledge and skills in designing and leading age-appropriate gross motor and fitness activities. Part of the training covered the modified PE curriculum, lesson plans, and new exercise stations. In vivo observation and hands-on practices were used to provide feedback and enhance the confidence of the PE teachers in delivering the modified curriculum independently. The training program was developed by the PE expert team and delivered by the lead author (Z.Z.) and graduate research assistants at each site.

#### 3.2.2. After-School Program

The ASP intervention was designed to provide an opportunity for additional MVPA and VPA during after-school hours that would not be available to students otherwise. It also aimed to engage parents in providing support for healthy habits at home, using a social media campaign. The ASP had three components:

(1) Provision of an extracurricular PA program and equipment. The schools agreed to provide the students with a 45 min PA program on school grounds twice a week that started immediately at the end of the school day. Portable equipment was also provided to the schools for implementing the program. The PE expert team developed fun and challenging group games, rhythmic aerobic dance routines, and rope jumping activities for the ASP. Physical fitness exercise stations with portable equipment were included as part of the ASP to target the development of speed, strength, endurance, agility, and balance. All seventh grade students were required to participate in the after-school program without exception. A trained PE teacher and assistant supervised the ASP.
(2) Parent engagement. A social media communication campaign was used to engage the seventh graders’ parents in creating a healthy home environment, with key messages on supporting healthy eating, discouraging excessive sedentary behavior (e.g., watching TV or playing computer games), and promoting adequate sleep. The parents also received updates on the status of their child’s growth and physical fitness. The campaign content was delivered to the parents twice a week via a group WeChat, administered by the research team.
(3) After school program teacher training. The PE teachers received a one-day training for delivering the ASP. The content and format of the training were similar to the SPE intervention.

#### 3.2.3. Control Conditions

Schools in the control group agreed to provide two 45 min PE classes each week. No training or PE equipment was provided to PE teachers in the control schools. The PE teachers were asked to conduct class activities as they used to in the past.

### 3.3. Study Measurements

Following a standardized protocol, we conducted objective assessments of the student participants’ physical fitness, cognitive functions, and PA, and collected parental reports of their child’s health-related behaviors and family support for healthy living at home, in order to evaluate the impacts of the intervention on the primary and secondary outcomes of the study. The data collection was performed by trained graduate research assistants from the first author’s institution, and occurred at the study sites at the beginning and end of the study. The graduate research assistants, who were blinded to the treatment conditions, completed a one-day measurement training under the supervision of the first and second authors (Z.Z. and Q.F.) prior to each data collection. The measures used in the study have been shown to be valid in Chinese adolescents. Table 4 provides a summary of the outcome measures along with information regarding the relevant validation studies.

The primary outcome of the study was physical fitness, defined as the body’s ability to perform at an optimal level in response to a physiological stress and manifested through six attributes (CRF or aerobic fitness, muscular strength, speed, agility, flexibility, and body composition) [46]. We used the Chinese National Students’ Physical Fitness and Health Standard, which is a field-based test battery, to assess each student’s physical fitness [23]. The measures included a 20 m multistage shuttle run (20 MSR) for CRF, a broad jump for lower limb muscle strength, 1 min of sit-ups for abdominal and lower back muscle strength, a 50 m run for speed, a *t*-test agility run for agility, sit and reach for flexibility, and body fat percentage for body composition. The 20 MSR has been shown to be highly correlated with peak consumption of oxygen, and is widely used as an indicator of CRF in youth [47,48,49]. The body fat percentage was measured by a body composition analyzer with a pediatric reference norm, following the procedure recommended by the manufacturer. We chose the national physical fitness test for this study to assess the broader effects of the proposed PA intervention that might not be captured by health-related fitness tests [50]. For example, speed and agility were not included in the health-related fitness tests, such as FitnessGram [51], but were targeted by the Chinese CHAMPS intervention. We also measured height and weight to calculate the BMI and BMI *z*-score for age and gender, following the standards recommended by the International Obesity Task Force [44].

We were also interested in exploring the effects of the intervention program on cognitive functions in this study. These were broadly defined as the ability to process, store, and utilize information by the central nervous system [12]. Previous studies have demonstrated that aerobic exercise interventions improve executive functioning in children [10]. This study provides data to test the different impacts on cognitive functions of varied amount of MVPA and VPA. We measured three different aspects of cognitive functioning—fluid intelligence, attention control, and executive function—in the study participants (see Table 4). Finally, the students completed a demographic survey (school grade and sex) and reported their pubertal stage using the pubertal development scale [52].

The parents of the students completed a survey to assess the availability of fruits and vegetables and support for physical activity and healthy eating at home, and reported their own height, weight, and levels of physical activity (minutes of moderate PA (MPA)/day and VPA/day) using the Rapid Assessment of Physical Activity Questionnaire [53,54]. The parents also reported their highest level of education and monthly family income. The surveys were brought home by the students. The completed surveys were returned to the PE teachers at each student’s school. 

### 3.4. Process Evaluation Measures

Accelerometers (Actigraph GT3X-Plus, Pensacola, FL, USA) were used to measure the amount of time spent each day in light, moderate, and vigorous activities and sedentary behavior [55]. Half of the students were randomly selected to wear the accelerometers on their right hip from the time of wake-up to bedtime for seven consecutive days. To be considered as a valid measure, the accelerometer must be worn at least 10 h/d for 4 days or more, including one weekend day. Activity counts were processed using Evenson’s cut-points, modified for Chinese children, using ActiLife6 software (version 6.11.5) [56,57].

We conducted a process evaluation to assess the fidelity of intervention implementation in both the intervention and control schools during the study. We collected information on the number of days that PE class, recess, and ASP were offered, and student attendance information at each study school. The PE assistant conducted a PE class observation twice during the study using five randomly selected students with the System for Observing Fitness Instruction Time (SOFIT) [58], which provided an evaluation of the quality of PE instruction and estimation of student participation in low, moderate, and vigorous activities in the class. The same five students also wore Polar Team2 Pro heart rate monitors (Polar Electro Oy, Kempele, Finland, Finland) to assess the activity intensity during the class. Finally, to evaluate if the intervention impacted student attitude and knowledge, all students completed a food habits and preference survey [59], nutrition knowledge test [60], the Physical Activity Questionnaire for Children [61], and a record of sedentary behavior at baseline and post-test [62].

### 3.5. Statistical Analysis

Because the proposed study was a cluster RCT, to obtain efficient and unbiased model estimates (and therefore unbiased estimates of the intervention effect), the analyses should account for variation due to schools, participants, and within-participant correlation due to repeated measures. Thus, the study participants were the units of analysis. Although a full parametric modeling approach (such as generalized linear mixed effect modeling) would lead to the most efficient estimates, the results might not be robust if the parametric assumptions were violated. Therefore, we proposed using the GEEs approach [63] for a 2 × 2 factorial design of the school PE intervention (yes, no) by the afterschool program intervention (yes, no) in order to test the research hypotheses in the study. Two random effects were included in the GEEs analyses: one to account for the correlation among students nested within the same school, and one to account for the two repeated measures of each outcome with the same student. School size (small, large), which is a fixed effect, was also included in the model. We additionally planned to explore the influence of covariates, including student gender, pubertal stage, parent education level, family income, and family support for PA and healthy eating. The GEEs approach is a semi-parametric technique for correlated data, which only requires specification of the relationship between the means of the outcomes and the associated predictors, and the estimation is robust to the assumed correlation structure. Model fit was assessed by residual diagnostics [63] to guide the best fit model. All statistical tests were two-sided with a significance level of ≥5% and statistical analysis system (SAS) 9.4 for Windows (SAS Institute, Cary, NC, USA) was used to perform the analysis.

## 4. Discussion

The findings from the Chinese CHAMPS study have the potential to address many questions and debates around school-based PA intervention research. First, the factorial design provides a much-needed opportunity to test the effect of a varied amount of, and intensity of, MVPA and VPA on physical fitness and cognitive functioning in Chinese middle school students. Of particular interest were the multi-level interventions that targeted the PE and afterschool policies, curriculum, and teacher training in Chinese middle schools, and a physical fitness focus that stressed VPA. However, the study design does not allow us to evaluate whether the changes in the study outcomes are the results of changes in the frequency of MVPA or the intensity of the activities of the PA intervention.

Second, the use of accelerometers allowed us to accurately measure the amount and intensity of PA and sedentary behavior. The impacts of VPA, a metabolically unique activity [64], on physical fitness and cognitive function are not well understood in school-age children and were evaluated with adjustment to MPA, light PA (LPA), sedentary behavior, and sleep in this study [65].

Third, there is an on-going debate around whether engaging in MVPA leads to reduction of MVPA or an increase in LPA or sedentary behavior during other times of the day, or on later days in the week [66]. While some studies found no cross-day changes in MVPA [67], others reported that children showed an increase in sedentary time during other times of the day after participating in MVPA [68]. With the seven-day accelerometry data, we can explore how the Chinese CHAMPS intervention influenced active time and sedentary time in school-age Chinese children across a timespan of seven days.

Fourth, the PA program is designed to reduce boredom and lack of interest, which are common barriers for participation in PA in school-age children [69]. Other researchers have shown that PA programs with a developmentally appropriate approach and fundamental movement skill training can lead to increases in long-term participation in sports and PA [70], gross motor skills [71,72], and physical fitness [73,74] in children and adolescents.

Fifth, the study examined whether the addition of an afterschool program could increase the dose of PA intervention and its effects on the outcome measures. Although afterschool interventions have been shown to improve physical fitness and body composition [75,76], the feasibility and benefits of combining them with a school-based PE intervention is not known.

Finally, China has a predominantly public co-education school system funded by central and local government that strongly influence local school policies, curricula, and teacher training. Findings from this study may provide much-needed information to develop policy recommendations that promote MVPA across all schools in China.

The high-stakes testing culture has traditionally overlooked the health values of PE, and in doing so endangered the wellbeing of Chinese school-age children [77,78]. Recently, rapid economic growth and urbanization in China has imparted new challenges and barriers for meeting PA recommendations and maintaining a healthy lifestyle [18,79]. However, evidence-based intervention strategies for Chinese schools are sparse at the present time. In response to the need to promote MVPA in Chinese schools, it is important to scrutinize the literature and validate study findings based on Western populations, as well as to address issues unique to PA promotion in the Chinese school-setting [22]. Lessons learned from this study may be informative to Chinese education officials in China and other developing countries who are facing many of the same issues in promoting PA in schools [80].

## 5. Conclusions

Meeting the recommendation of ≥1 h/d of MVPA in children and adolescents is challenging in both developed and developing countries [39,81]. The design of the Chinese CHAMPS study incorporated current evidence and best practice to examine the effects of increasing the dose of PA in middle school students. Findings from the study potentially shed light on many of the issues and challenges encountered in school-based PA intervention research.

## Figures and Tables

**Table 1:** Time allocation of physical activity (PA) and vigorous physical activity (VPA) in the 2 × 2 experimental conditions.

	Physical Education Class	Daily Class Recess	Afterschool Physical Activity Program
Arm 1: SPE (210 min/week; ≥105 min of VPA/week)	45 min × 3 days/week (135 min/week; 68 min of VPA/week)	15 min × 5 days/week (75 min/week; 38 min of VPA/week)	
Arm 2: ASP (225 min/week; ≥45 min of VPA/week)	45 min × 3 days/week (135 min/week)		45 min × 2 days/week (90 min/week; 45 min of VPA/week)
Arm 3: SPE + ASP (300 min/week; ≥150 min of VPA/week)	45 min × 3 days/week (135 min/week; 68 min of VPA/week)	15 min × 5 days/week (75 min/week; 38 min of VPA/week)	45 min × 2 days/week (90 min/week; 45 min of VPA/week)
Arm 4: Control (90 min/week)	45 min × 2 day/week (90 min/week)		

SPE: School Physical Education policy and curriculum intervention; ASP: afterschool physical activity program. The goal is to introduce vigorous physical activities in ≥50% of the allocated time for physical activity intervention. No change will be introduced in Physical Education Classes in ASP and the Control conditions.

**Table 2:** Functions targeted by physical functional training and types of exercises.

Targeted Function	Exercises
Movement preparation	Glute activation (mini-band), dynamic stretching, movement integration/dissociation, and neural activation.
Plyometric (stretch-shortening cycle)	Movement pattern (catch-throw, push-up, jump, hop, bound), movement direction (linear, lateral and rotation with vertical or horizontal), movement initiation (no-counter movement, counter movement, double contact, and continuous), such as lateral bounding/lateral hurdle hopping.
Movement skills	Motor programing and skill application (sport specific), with resisted, assisted, and combination.
Speed and agility training	Fundamental speed drills transferred to maximal acceleration, velocity, and speed-endurance training, multi-directional speed (shuffle, crossover, change of direction, and drop-step), various agility drills utilizing pre-marked distances (lines), cones, and ladders.
Strength and power training	Movement based training (movement patterns, multiple joint movement, multi-planar, eccentric, concentric, isometric); pillar strength (plank, glute bridge, lateral pillar) Movement types: upper push and pull, lower push and pull, rotational, and total body.
Energy system development	Repetition sprints, interval sprints, fartlek training in anaerobic conditioning. Long-duration and moderate-intensity training, short-duration and high-intensity training in the aerobic endurance training.Five-phases: base, interval, linear, multi-direction, sport specific.
Regeneration and recovery	Stretching techniques, soft tissue.

**Table 3:** Description of intervention activities.

School Health-Related Fitness Promotion	After-School Program
Intervention activities	
**Physical activity policies and environment** Provision of PE classes at a minimum of 3 days/week and daily 15 min recess. Provision of portable exercise equipment.	**Physical activity policies and environment** Provision of bi-weekly 45 min afterschool PA program. Provision of portable exercise equipment.
**PE Curriculum** Redesign of PE curriculum (introduction of exercise stations, and modification of games and sport activities) and provision of weekly lesson plans. Development of a 15 min recess rhythmic aerobic movement routine. Use of Adolescent Fitness and Health Handbook to develop fitness and nutrition knowledge and skills for inclement weather days. Bi-weekly text messages with WeChat to deliver health and nutrition tips to students on their mobile phones.	**After School Curriculum** Development of 45 min physical activity program with group games and exercise stations. Adolescent Fitness and Health Handbook to develop fitness and nutrition knowledge and skills for inclement weather days. Bi-weekly text messages with WeChat to deliver updates to parents on adolescent’s growth and changes in physical fitness status, and information on healthy eating, physical activity, and obesity on their mobile phones.
**Staff Development** 2 days teacher training on adolescent growth and development, design of age-appropriate gross motor programs and physical activities, and pedagogical methods and instructional strategies. In vivo observation and hand-on practices with experts.	**Staff Development** 1 day teacher training on adolescent growth and development, design of age-appropriate gross motor programs and physical activities, and pedagogical methods and instructional strategies. In vivo observation and hand-on practices with experts.
Intervention delivery	
Middle school PE teachers. Research assistants.	Research staff. Middle school staff. Adolescents’ parents.
Process evaluation	
Periodic monitoring and feedback using System for Observing Fitness Instruction Time. Monitoring of activity intensity by heart rate monitors during PE classes. Monthly audit on number of PE classes and PA during recess offered each week. Quizzes on health and nutrition knowledge.	Periodic monitoring and feedback using System for Observing Fitness Instruction Time. Monitoring of activity intensity by heart rate monitors during PE classes.

Abbreviation: PE, physical education; PA, physical activity.

**Table 4:** Description of study outcome measurement.

Measure(s)	Purpose	Instrumentation
Student Outcome Measures		
Physical fitness	To measure levels of physical fitness [43].	The 20-m Multistage Shuttle Run for aerobic fitness; broad jump for low limbs muscle strength; 1 min sit-up for core muscle strength; *t*-test agility run for agility; 50 m run for speed; sit-and-reach test for flexibility; body fat percent for body composition.
Weight and height	To calculate BMI (weight in kg/height in m^2^) and the BMI *z*-score as a proxy measure of adiposity [44].	Weight by digital electronic scale (MC-180MA, Tanita Corporation, Tokyo, Japan); height by stadiometer (Seca 213, Seca Deutschland, Hamburg, Germany).
Waist circumference and waist-to-height ratio	To estimate central adiposity [45].	The circumference measured at the narrowest point of the midsection by a plastic non-elastic tape.
Cognitive functions	To measure levels of fluid intelligence, attention control, and executive function.	Raven’s Standard Progressive Matrices for fluid intelligence; the Adolescent Attention Test for attention control (attention allocation, attention span, attention stability and attention switching); the Flanker Tests for executive function.

Abbreviation: BMI, body mass index.

## Abbreviations

ASP	After-school program
CRF	Cardiorespiratory fitness
LPA	Light physical activity
MPA	Moderate physical activity
MVPA	Moderate to vigorous physical activity
PA	Physical activity
PFT	Physical function training
SPE	School physical education intervention
VPA	Vigorous physical activity

## References

[1] Katzmarzyk P.T., Barreira T.V., Broyles S.T., Champagne C.M., Chaput J.P., Fogelholm M., Hu G., Johnson W.D., Kuriyan R., Kurpad A. (2015). Physical activity, sedentary time, and obesity in an international sample of children. Med. Sci. Sports Exerc..

[2] World Health Organization (2010). Global Recommendations on Physical Activity for Health.

[3] Twisk J.W.R. (2001). Physical activity guidelines for children and adolescents. Sports Med..

[4] Hay J., Maximova K., Durksen A., Carson V., Rinaldi R.L., Torrance B., Ball G.D.C., Majumdar S.R., Plotnikoff R.C., Veugelers P. (2012). Physical activity intensity and cardiometabolic risk in youth. Arch. Pediatr. Adolesc. Med..

[5] Ruiz J.R., Wärnberg J., Sjöström M., Ortega F.B., Rizzo N.S., Hurtig-Wennlöf A. (2006). Relations of total physical activity and intensity to fitness and fatness in children: The European youth heart study (erratum: 2009 feb., v. 89, no. 2, p. 656.). Am. J. Clin. Nutr..

[6] Carson V., Rinaldi R.L., Torrance B., Maximova K., Ball G.D.C., Majumdar S.R., Plotnikoff R.C., Veugelers P., Boulé N.G., Wozny P. (2014). Vigorous physical activity and longitudinal associations with cardiometabolic risk factors in youth. Int. J. Obes..

[7] Tremblay M.S., LeBlanc A.G., Kho M.E., Saunders T.J., Larouche R., Colley R.C., Goldfield G., Connor Gorber S. (2011). Systematic review of sedentary behaviour and health indicators in school-aged children and youth. Int. J. Behav. Nutr. Phys. Act..

[8] Sallis J.F. (1993). Epidemiology of physical activity and fitness in children and adolescents. Crit. Rev. Food Sci. Nutr..

[9] Carson V., Hunter S., Kuzik N., Gray C.E., Poitras V.J., Chaput J.P., Saunders T.J., Katzmarzyk P.T., Okely A.D., Connor Gorber S. (2016). Systematic review of sedentary behaviour and health indicators in school-aged children and youth: An update. Appl. Physiol. Nutr. Metab..

[10] Tomporowski P.D., Lambourne K., Okumura M.S. (2011). Physical activity interventions and children’s mental function: An introduction and overview. Prev. Med..

[11] Martin A., Saunders D.H., Shenkin S.D., Sproule J. (2014). Lifestyle intervention for improving school achievement in overweight or obese children and adolescents. Cochrane Database Syst. Rev..

[12] Donnelly J.E., Hillman C.H., Castelli D., Etnier J.L., Lee S., Tomporowski P., Lambourne K., Szabo-Reed A.N. (2016). Physical activity, fitness, cognitive function, and academic achievement in children: A systematic review. Med. Sci. Sports Exerc..

[13] The World Health Organization (2007). Promoting Physical Activity in Schools: An Important Element of a Health-Promoting School. WHO Information Series on School Health.

[14] Langford R., Bonell C., Jones H., Campbell R. (2015). Obesity prevention and the health promoting schools framework: Essential components and barriers to success. Int. J. Behav. Nutr. Phys. Act..

[15] Dobbins M., Husson H., DeCorby K., LaRocca R.L. (2013). School-based physical activity programs for promoting physical activity and fitness in children and adolescents aged 6 to 18. Cochrane Libr..

[16] Zhang J., Seo D.C., Kolbe L., Middlestadt S., Zhao W. (2012). Associated trends in sedentary behavior and BMI among Chinese school children and adolescents in seven diverse Chinese provinces. Int. J. Behav. Med..

[17] Chen Y., Zheng Z., Yi J., Yao S. (2014). Associations between physical inactivity and sedentary behaviors among adolescents in 10 cities in China. BMC Public Health.

[18] Lu C., Stolk R.P., Sauer P.J., Sijtsma A., Wiersma R., Huang G., Corpeleijn E. (2017). Factors of physical activity among Chinese children and adolescents: A systematic review. Int. J. Behav. Nutr. Phys. Act..

[19] He Q.Q., Wong T.W., Du L., Jiang Z.Q., Yu T.S., Qiu H., Gao Y., Liu W.J., Wu J.G. (2011). Physical activity, cardiorespiratory fitness, and obesity among Chinese children. Prev. Med..

[20] The Physical Fitness and Health Research of Chinese School Students Study Group (2008). 2005 Reports on the Physical Fitness and Health Research of Chinese School Students.

[21] Ren H., Zhou Z., Liu W., Wang X., Yin Z. (2017). Excessive homework, inadequate sleep, physical inactivity and screen viewing time are major contributors to high paediatric obesity. Acta Paediatr..

[22] National Institute of Education Science (2009). The Survey of the Current Status of Physical Education in Primary and Secondary Schools in China (2006.9–2008.10).

[23] Ministry of Education of the China (2014). Chinese National Student Physical Health Standard.

[24] Wang Y., Cai L., Wu Y., Wilson R.F., Weston C., Fawole O., Bleich S.N., Cheskin L.J., Showell N.N., Lau B.D. (2015). What childhood obesity prevention programmes work? A systematic review and meta-analysis. Obes. Rev..

[25] Wareham N.J., Hennings S.J., Byrne C.D., Hales C.N., Prentice A.M., Day N.E. (1998). A quantitative analysis of the relationship between habitual energy expenditure, fitness and the metabolic cardiovascular syndrome. Br. J. Nutr..

[26] Verstraeten R., Roberfroid D., Lachat C., Leroy J.L., Holdsworth M., Maes L., Kolsteren P.W. (2012). Effectiveness of preventive school-based obesity interventions in low- and middle-income countries: A systematic review. Am. J. Clin. Nutr..

[27] Van Sluijs E.M.F., McMinn A.M., Griffin S.J. (2007). Effectiveness of interventions to promote physical activity in children and adolescents: Systematic review of controlled trials. BMJ.

[28] Lander N., Eather N., Morgan P.J., Salmon J., Barnett L.M. (2017). Characteristics of teacher training in school-based physical education interventions to improve fundamental movement skills and/or physical activity: A systematic review. Sports Med..

[29] Waters E., de Silva-Sanigorski A., Burford B.J., Brown T., Campbell K.J., Gao Y., Armstrong R., Prosser L., Summerbell C.D. (2011). Interventions for preventing obesity in children. Cochrane Database Syst. Rev..

[30] Sallis J.F.O.N., Fisher E.B., Glanz K.R.B.K., Viswanath K. (2008). Ecological models of health behavior. Health Behavior and Health Education: Theory, Research and Practice.

[31] Conroy D.E., Elliot A.J., Coatsworth J.D. (2007). Competence motivation in sport and exercise: The hierarchical model of achievement motivation and self-determination theory. Intrinsic Motivation and Self-Determination in Exercise and Sport.

[32] Ministry of Education of the China (2011). National Physical Education and Health Education Curriculum Standards-2011.

[33] Ministry of Education of the China (2004). Ministry of Education Notification on Time Recommendation for Physical Education Class in Elementary, Middle and High Schools.

[34] Gutin B., Barbeau P., Yin Z. (2004). Exercise interventions for prevention of obesity and related disorders in youths. Quest.

[35] Davis C.L., Pollock N.K., Waller J.L., Allison J.D., Dennis B.A., Bassali R., Meléndez A., Boyle C.A., Gower B.A. (2012). Exercise dose and diabetes risk in overweight and obese children: A randomized controlled trial. JAMA.

[36] Barbeau P., Johnson M.H., Howe C.A., Allison J., Davis C.L., Gutin B., Lemmon C.R. (2007). Ten months of exercise improves general and visceral adiposity, bone, and fitness in black girls. Obesity.

[37] Laguna M., Ruiz J.R., Lara M.T., Aznar S. (2013). Recommended levels of physical activity to avoid adiposity in Spanish children. Pediatr. Obes..

[38] Wittmeier K.D., Mollard R.C., Kriellaars D.J. (2008). Physical activity intensity and risk of overweight and adiposity in children. Obesity.

[39] Rizzo N.S., Ruiz J.R., Hurtig-Wennlöf A., Ortega F.B., Sjöström M. (2007). Relationship of physical activity, fitness, and fatness with clustered metabolic risk in children and adolescents: the European youth heart study. J. Pediatr..

[40] Pavey T.G., Gilson N.D., Gomersall S.R., Clark B., Trost S.G. (2017). Field evaluation of a random forest activity classifier for wrist-worn accelerometer data. J. Sci. Med. Sport.

[41] Zhou Z. (2017). Physical function training and physical fitness in middle school students. Theory and Practice of Physical Function Training for Middle School Students.

[42] Yin J. (2017). An overview of training idea and content system of physical function training. Teach. Phys. Educ..

[43] The State General Administration of Sports (2003). Chinese National Measurement Standards on People’s Physical Fitness.

[44] Cole T.J., Bellizzi M.C., Flegal K.M., Dietz W.H. (2000). Establishing a standard definition for child overweight and obesity worldwide: International survey. BMJ.

[45] Taylor R.W., Jones I.E., Williams S.M., Goulding A. (2000). Evaluation of waist circumference, waist-to-hip ratio, and the conicity index as screening tools for high trunk fat mass, as measured by dual-energy X-ray absorptiometry, in children aged 3–19 y. Am. J. Clin. Nutr..

[46] Malina R.M. (2001). Physical activity and fitness: Pathways from childhood to adulthood. Am. J. Hum. Biol..

[47] Tomkinson G.R., Leger L.A., Olds T.S., Cazorla G. (2003). Secular trends in the performance of children and adolescents (1980–2000): An analysis of 55 studies of the 20 m shuttle run test in 11 countries. Sports Med..

[48] Eisenmann J.C., Welk G.J., Ihmels M., Dollman J. (2007). Fatness, fitness, and cardiovascular disease risk factors in children and adolescents. Med. Sci. Sports Exerc..

[49] Research CIfA (1999). Fitnessgram Test Administration Manual.

[50] Pate R.R. (1983). A new definition of youth fitness. Phys. Sportsmed..

[51] The Cooper Institute (2004). Fitnessgram Test Administration Manual.

[52] Sun Y., Tao F.B., Su P.Y. (2012). Self-assessment of pubertal tanner stage by realistic colour images in representative chinese obese and non-obese children and adolescents. Acta Paediatr..

[53] (2006). Rapid Assessment of Physical Activity (RAPA). http://depts.washington.edu/hprc/rapa.

[54] Topolski T.D., LoGerfo J., Patrick D.L., Williams B., Walwick J., Patrick M.B. (2006). The rapid assessment of physical activity (RAPA) among older adults. Prev. Chronic Dis..

[55] Colley R., Connor Gorber S., Tremblay M.S. (2010). Quality control and data reduction procedures for accelerometry-derived measures of physical activity. Health Rep..

[56] Zhu Z., Chen P., Zhuang J. (2013). Intensity classification accuracy of accelerometer-measured physical activities in Chinese children and youth. Res. Q. Exerc. Sport.

[57] Zhu Z., Chen P., Zhuang J. (2013). Predicting Chinese children and youth’s energy expenditure using actigraph accelerometers: A calibration and cross-validation study. Res. Q. Exerc. Sport.

[58] McKenzie TL S.J., Nader P.R. (1991). SOFIT: System for observing fitness instruction time. J. Teach. Phys. Educ..

[59] Johnson F., Wardle J., Griffith J. (2002). The adolescent food habits checklist: Reliability and validity of a measure of healthy eating behaviour in adolescents. Eur. J. Clin. Nutr..

[60] Turconi G., Celsa M., Rezzani C., Biino G., Sartirana M.A., Roggi C. (2003). Reliability of a dietary questionnaire on food habits, eating behaviour and nutritional knowledge of adolescents. Eur. J. Clin. Nutr..

[61] Crocker P.R.E., Bailey D.A., Faulkner R.A., Kowalski K.C., McGrath R. (1997). Measuring general levels of physical activity: Preliminary evidence for the physical activity questionnaire for older children. Med. Sci. Sports Exerc..

[62] Duan J., Hu H., Wang G., Arao T. (2015). Study on current levels of physical activity and sedentary behavior among middle school students in Beijing, China. PLoS ONE.

[63] Diggle P.J., Liang K.Y., Zeger S.L. (1994). Analysis of Longitudinal Data.

[64] Westerterp K.R. (2003). Impacts of vigorous and non-vigorous activity on daily energy expenditure. Proc. Nutr. Soc..

[65] Fanning J., Porter G., Awick E., Ehlers D., Roberts S., Cooke G., Burzynska A., Voss M., Kramer A., McAuley E. (2017). Replacing sedentary time with sleep, light, or moderate-to-vigorous physical activity: Effects on self-regulation and executive functioning. J. Behav. Med..

[66] Reilly J.J. (2011). Can we modulate physical activity in children?. Int. J. Obes..

[67] Goodman A., Mackett R.L., Paskins J. (2011). Activity compensation and activity synergy in British 8–13 year olds. Prev. Med..

[68] Ridgers N.D., Timperio A., Cerin E., Salmon J. (2014). Compensation of physical activity and sedentary time in primary school children. Med. Sci. Sports Exerc..

[69] Flynn M.A.T., McNeil D.A., Maloff B., Mutasingwa D., Wu M., Ford C., Tough S.C. (2006). Reducing obesity and related chronic disease risk in children and youth: A synthesis of evidence with ‘best practice’ recommendations. Obes. Rev..

[70] Standage M., Duda J.L., Ntoumanis N. (2005). A test of self-determination theory in school physical education. Br. J. Educ. Psychol..

[71] Morgan P.J., Barnett L.M., Cliff D.P., Okely A.D., Scott H.A., Cohen K.E., Lubans D.R. (2013). Fundamental movement skill interventions in youth: A systematic review and meta-analysis. Pediatrics.

[72] Logan S.W., Robinson L.E., Wilson A.E., Lucas W.A. (2012). Getting the fundamentals of movement: A meta-analysis of the effectiveness of motor skill interventions in children. Child Care Health Dev..

[73] Wright M.D., Portas M.D., Evans V.J., Weston M. (2015). The effectiveness of 4 weeks of fundamental movement training on functional movement screen and physiological performance in physically active children. J. Strength Cond. Res..

[74] Lubans D.R., Morgan P.J., Cliff D.P., Barnett L.M., Okely A.D. (2010). Fundamental movement skills in children and adolescents: Review of associated health benefits. Sports Med..

[75] Atkin A., Gorely T., Biddle S., Cavill N., Foster C. (2011). Interventions to promote physical activity in young people conducted in the hours immediately after school: A systematic review. Int. J. Behav. Med..

[76] Yin Z., Moore J.B., Johnson M.H., Vernon M.M., Gutin B. (2012). The impact of a 3-year after-school obesity prevention program in elementary school children. Child Obes..

[77] Commentary B.K. (2003). Physical and mental health of contemporary Chinese children. J. Fam. Econ. Issues.

[78] Lin J., Chen Q. (1995). Academic pressure and impact on students’ development in China. McGill J. Educ..

[79] Song Y., Wang H.J., Dong B., Ma J., Wang Z., Agardh A. (2016). 25-year trends in gender disparity for obesity and overweight by using WHO and IOTF definitions among Chinese school-aged children: A multiple cross-sectional study. BMJ Open.

[80] Barbosa Filho V.C., Minatto G., Mota J., Silva K.S., de Campos W., Lopes Ada S. (2016). Promoting physical activity for children and adolescents in low- and middle-income countries: An umbrella systematic review: A review on promoting physical activity in LMIC. Prev. Med..

[81] Andersen L.B., Harro M., Sardinha L.B., Froberg K., Ekelund U., Brage S., Anderssen S.A. (2006). Physical activity and clustered cardiovascular risk in children: A cross-sectional study (the European youth heart study). Lancet.

